# A Peculiar Case of Closure of Jaws

**Published:** 1872-10

**Authors:** J. E. Brooks


### ARTICLE X.

### *A Peculiar Case of Closure of Jaws.*

BY J. E. BROOKS, M. R. C. S., ETC.

Alexander D——, aged twenty-one, a miner, applied to me for advice on February 1st, 1871. He states that when he was about six years old he had ulcerated gums on the left side, and a bandage that was placed around his head caused them and his cheek to grow together. He has never since been able to open his mouth more than a quarter of an inch. He has worked at his trade at intervals, but was often unable to do so from weakness, consequent upon taking little solid food. Upon examination I found what he said to be correct, the parts being firmly adherent, and the jaws almost closed. The temporo-maxillary articulation was not affected, which is somewhat singular, considering the small amount of movement that had taken place in it. I placed him under the influence of chloroform, and divided the rigid band of tissue, which gave him temporary relief; cicatrization, however, fixing it more firmly than before. The same operation was subsequently performed by myself and several other surgeons to whom he applied, with the same unfortunate result.

At the commencement of July the patient again presented himself, being very anxious to be cured; and in the meantime, having given the case some consideration, it occurred to me that I might meet with more success if I fixed his mouth open during the healing process. I accordingly administered chloroform to him, and, having cut through the cicatrix, I placed between his teeth a piece of wood about an inch thick. This he allowed to remain about twenty-four hours; but the pain from it was so severe that he removed it himself. He subsequently kept a piece in during the night and at intervals during the day.

On July 22nd he returned to his work, and has followed his employment ever since.

Feb. 20th, 1872. He can now open his mouth at least an inch, and has increased in weight about 20 pounds. To show the complete success of the operation, I may say that I have since removed the lower wisdom tooth on the affected side.

I have purposely waited before publshing the case to see that he had no recurrence of the symptoms.—*London Lancet.*

